# Safety of BRAF+MEK Inhibitor Combinations: Severe Adverse Event Evaluation

**DOI:** 10.3390/cancers12061650

**Published:** 2020-06-22

**Authors:** Tomer Meirson, Nethanel Asher, David Bomze, Gal Markel

**Affiliations:** 1 Ella Lemelbaum Institute for Immuno-oncology, Sheba Medical Center, Ramat-Gan 526260, Israel; tomermrsn@gmail.com (T.M.); asher.nati@gmail.com (N.A.); 2 Azrieli Faculty of Medicine, Bar-Ilan University, Safed 1311502, Israel; 3 Sackler Faculty of Medicine, Tel Aviv University, Tel Aviv 6997801, Israel; davidbomze@gmail.com; 4 Department of Clinical Microbiology and Immunology, Sackler Faculty of Medicine, Tel Aviv University, Tel-Aviv 6997801, Israel

**Keywords:** BRAF and MEK inhibitors, melanoma, pharmacovigilance, disproportionality analysis

## Abstract

**Aim:** The selective BRAF and MEK inhibitors (BRAFi+MEKi) have substantially improved the survival of melanoma patients with BRAF V600 mutations. However, BRAFi+MEKi can also cause severe or fatal outcomes. We aimed to identify and compare serious adverse events (sAEs) that are significantly associated with BRAFi+MEKi. **Methods:** In this pharmacovigilance study, we reviewed FDA Adverse Event Reporting System (FAERS) data in order to detect sAE reporting in patients treated with the combination therapies vemurafenib+cobimetinib (V+C), dabrafenib+trametinib (D+T) and encorafenib+binimetinib (E+B). We evaluated the disproportionate reporting of BRAFi+MEKi-associated sAEs. Significant associations were further analyzed to identify combination-specific safety signals among BRAFi+MEKi. **Results:** From January 2018 through June 2019, we identified 11,721 sAE reports in patients receiving BRAFi+MEKi. Comparison of BRAFi+MEKi combinations demonstrates that skin toxicities, including Stevens–Johnson syndrome, were disproportionally reported using V+C, with an age-adjusted reporting odds ratio (adj. ROR) of 3.4 (95%CI, 2.9–4.0), whereas fever was most significantly associated with D+T treatment with an adj. ROR of 1.9 (95%CI, 1.5–2.4). Significant associations using E+B treatment include peripheral neuropathies (adj. ROR 2.7; 95%CI, 1.2–6.1) and renal disorders (adj. ROR 4.1; 95%CI, 1.3–12.5). Notably, we found an increase in the proportion of Guillain–Barré syndrome reports (adj. ROR 8.5; 95%CI, 2.1–35.0) in patients administered E+B. **Conclusion:** BRAFi+MEKi combinations share a similar safety profile attributed to class effects, yet concomitantly, these combinations display distinctive effects that can dramatically impact patients’ health. Owing to the limitations of pharmacovigilance studies, some findings warrant further validation. However, the possibility of an increased risk for these events should be considered in patient care.

## 1. Introduction

Combination therapy with BRAF plus MEK inhibitors (BRAFi+MEKi) has transformed the treatment landscape and improved the clinical outcomes of patients with melanoma harboring *BRAF*^v600^ mutations [1,2,3,4,5,6,7]. Treatment with BRAFi+MEKi produces response rates of 68% for vemurafenib+cobimetinib (V+C) [1], 67% for dabrafenib+trametinib (D+T) [2] and 63% for encorafenib+binimetinib (E+B) [8] in BRAF-mutated metastatic melanoma. Landmark data showed 3 year overall survival rates of 38.5% for V+C [3,4], 45% for D+T [6] and 47% for E+B [8]. However, despite similar inclusion criteria in general, comparison of the clinical outcomes cannot be made directly since some criteria differed between trials—for example, variable definitions of brain metastasis control [9]. Moreover, the studies show significant differences in patient populations with poor prognostic markers, such as elevated serum LDH [9].

The safety profiles of BRAFi+MEKi monotherapy and combination therapies were evaluated during the confirmatory trials [1,2,3,4,5,6,10,11,12,13,14,15,16,17,18]. Some adverse reactions can be ascribed both to BRAFi and MEKi class effects, including gastrointestinal side effects, increases in transaminases and cutaneous toxicities [19]; others are attributed to class-specific reactions such as squamous cell carcinomas (SCCs) and arthralgia in BRAFi [19,20,21] compared to ocular, edema and cardiovascular toxicities in MEKi therapy [22,23]. The addition of MEKi to BRAFi was associated with higher risk for developing various adverse events (AE); however, the co-administration of MEKi may mitigate some of the toxicities of BRAFi induced by ‘paradoxical activation’ of the mitogen-activated protein kinase (MAPK) pathway in BRAF wild-type cells [1,2,6,24]. In patients treated with the combination therapy, almost all developed AEs of any grade; severe adverse events (sAEs) occurred in 34% for V+C, 43% for D+T and 34% for E+B [19]. Combination-specific side effects include photosensitivity for V+C and fever for D+T, which are attributed to vemurafenib and dabrafenib, as they were already identified in BRAFi monotherapy trials [15,16,25]. However, combination-specific side effects for E+B are less clear. Nevertheless, encorafenib was associated with increased incidences of transient Bell’s palsy which also occurred with the respective combination [5,18].

Considering the similar clinical efficacy demonstrated by the three BRAFi+MEKi combinations, the therapeutic decision often relies on the safety profile that characterizes each of the combinations. However, despite numerous studies comparing different agents, there are no head-to-head clinical trials comparing the different combinations, and most safety data originate from the confirmatory phase III trials [19]. Moreover, there are no studies directly comparing the safety profile of all the combinations, and instead, are either comparing only between two products (e.g., vemurafenib versus dabrafenib) [26,27,28,29,30,31], focusing on specific adverse events [26,28,29,30,31,32,33,34], or based on confirmatory studies alone [19]. Defining and characterizing BRAFi+MEKi combination-specific events is a crucial issue for patient safety, especially given that a large proportion of patients are expected to develop severe toxicities. Here, we aimed to characterize and compare sAEs of BRAFi+MEKi captured in the real-world setting by performing a large-scale post-marketing surveillance using the FAERS database.

## 2. Methods

### 2.1. Study Design and Data Sources

The observational, retrospective, pharmacovigilance study is based on reports linked to suspect drugs in the FDA Adverse Events Reporting System (FAERS) [35,36]. The FAERS serves as the FDA’s repository of safety reports submitted by patients, health care professionals and pharmaceutical companies, and currently contains over 18 million reports [37]. Since the FAERS is a publicly available and anonymized database, institutional review board approval and informed consent was waived.

### 2.2. Procedures

This study included serious events leading to death, life-threatening conditions, disability, or hospitalization from January 2018 to December 2019. Despite available reports before 2018, this period was used to compare the reports of three BRAFi+MEKi combinations during the same timeframe. Of note, reports of AEs associated with E+B started in 2018. Adverse events were classified according to the Medical Dictionary for Regular Activities (MedDRA), which organizes terms into a hierarchy [38]. Duplicate cases were removed and general AEs such as ‘off-label use’ were excluded. Adverse events specific to combination therapy in the analysis were those notified as suspected to be caused by both BRAFi and MEKi as primary or secondary suspects. Additional administrative information was extracted for each report, including patient characteristics (sex and age); country of origin; drugs (dosage regimen, start and end dates of administration); indication for the drug; reactions (onset date, outcome, and response to rechallenge and dechallenge).

### 2.3. Statistical Analysis

Disproportionality analysis using the ‘case-control’ approach allows assessing whether the reporting rate of AEs is differentially elevated relative to expected rates of reporting in a pharmacovigilance database [39,40]. Disproportionate reporting rates of AEs could be indicative of potential risks associated with a particular agent or drug combination. To compare treatment-induced toxicities among different BRAFi+MEKi regimens, we also performed a disproportionate analysis between the combinations as a case–noncase study (e.g., V+C versus D+T and E+B). Standard measures of disproportionality include the information component (IC) when comparing to all the records in the dataset and the reporting odds ratio (ROR) when comparing between different treatment subgroups [41,42]. Thus, the latter analysis (ROR) involves only patients with BRAFi+MEKi and the rest of the database is excluded. The IC, given by a Bayesian confidence propagation neural network [41], and ROR, a point estimate calculated as the observed odds ratio [43], are widely used and are currently employed by various reporting agencies, as well as by the World Health Organization (WHO) [41,44,45].

The decision rule for a potential signal generation was based on a positive IC value (>0), a minimum number of reactions of 3, and a threshold of 0.05 for the Bayesian version of the false discovery rate (FDR) [46]. This analysis was performed on high-level group terms of drug-specific adverse events versus the full database (Supplementary Table S1). To compare reactions between BRAFi+MEKi combinations that are also disproportionally reported in the full database, we used only AEs linked to the identified signals above. In the ROR analysis, the signal-generation method was based on mid-*p*-values, as proposed by Ahmed et al. [47], and a signal was considered significant if ROR > 1 and the corrected *p*-value for multiple testing using the Benjamini–Hochberg (BH) correction was <0.05 [48]. Only combinations with a minimum amount of 0.5% reports were selected, corresponding to five reports in the smallest combination regimen (E+B). Given the considerable overlap between symptoms for various causes as well as redundancies in reporting AEs, we considered events for further evaluation if they were also significant in associated MedDRA terms. Since relevant medical information such as cancer staging was absent and therefore could not be accounted for, death and neoplasm occurrences as AEs were excluded from subsequent analysis. The disproportionality analysis and the age-adjusted ROR analysis were performed using the PhViD [49] and epiR [50] packages, respectively, in the R statistical computing environment (v3.5.2) [51].

## 3. Results

### 3.1. Patient Characteristics

The FAERS database included 11,721 reports of sAEs among patients receiving V+C (2345), D+T (8411) and E+B (965), corresponding to the time period (January 2018 to December 2019) (Table 1). The total number of sAEs reported in the FAERS was 3,285,265. Patient characteristics, drug indications, and country of origin are shown in Table 1.

### 3.2. Toxicity Profile of BRAFi and MEKi Compared to the Full Database

In order to compare reactions between different BRAFi+MEKi combinations that are also differentially elevated compared to other drugs, we first performed a disproportionality analysis compared to the background (excluding fatal outcomes and neoplasm-related events). The most common sAE reports in patients exposed to V+C were epidermal and dermal conditions with 311 (13.3%) cases (IC = 2.1; FDR < 10^−130^), followed by general systems disorders not elsewhere classified (NEC) with 249 (10.6%) cases (IC = 0.42; FDR < 10^−6^) (Supplementary Table S1). For patients treated with D+T, the most frequent sAE reports were general systems disorders NEC with 890 (10.6%) cases (IC = 0.45; FDR < 10^−20^) and body temperature conditions with 496 (5.9%) cases (IC = 2.28; FDR < 10^−258^). For patients treated with E+B, the most common sAE reports were general systems disorders with 116 (12.0%) cases (IC = 0.74; FDR < 10^−8^), followed by gastrointestinal signs and symptoms with 68 (7.0%) cases (IC = 1.22; FDR < 10^−12^).

### 3.3. Toxicity Profile of BRAFi and MEKi Combinations

**Figure 1:**
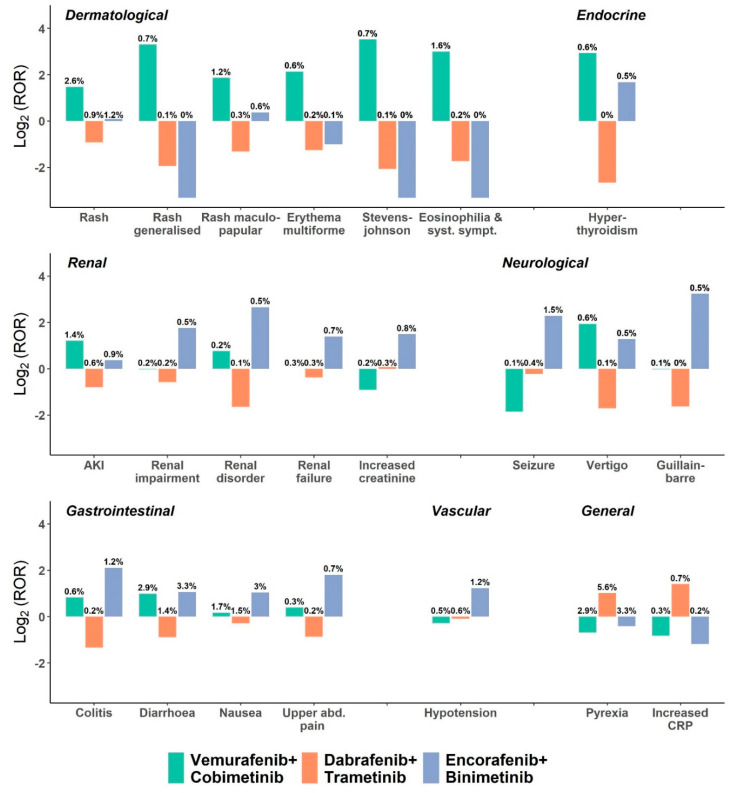
Comparison of serious adverse events (detected as signals) reported for BRAK-MEK inhibitors. The disproportionality analysis included serious adverse events leading to death, life-threatening conditions, disability, or hospitalization from January 2018 to June 2019. Adverse events associated with BRAFi+MEKi as primary or secondary suspects, which were also disproportionally reported compared to the full database, are displayed. The proportion of the events is shown on top of the bars. A constant of 0.1 was added to all ROR values to avoid bias of near zero values. AKI—acute kidney injury. CRP—C-reactive protein.

The reported frequencies of sAEs for the three BRAFi+MEKi combinations can be compared directly using ROR analysis. The detailed ROR estimates of V+C, D+T, and E+B for sAEs detected as signals are listed in Supplementary Table S2. To facilitate the analysis and interpretation of the data, the ROR for each identified sAE was compared: the respective shifts in BRAFi+MEKi ROR, expressed as an increase or decrease in a logarithmic scale, are displayed in Figure 1. ROR of dermatological side effects including rash, generalized rash, maculo-papular rash, erythema multiforme and rash with eosinophilia and systemic symptoms were considerably higher in V+C compared to D+T and E+B, whereas pyrexia and elevated C-reactive protein (CRP) were disproportionally higher in D+T compared to V+C and E+B. Furthermore, gastrointestinal events including colitis, diarrhea, nausea and upper abdominal pain and renal side effects including renal disorder/impairment/failure and increased creatinine were disproportionally higher in E+B compared to other BRAFi+MEKi combinations. Acute kidney injury (AKI) was higher in V+C, compared to E+B; however, both were considerably higher than D+T. Notably, Guillain–Barré syndrome (GBS) and seizures were significantly higher in E+B compared to V+C and D+T.

We conducted further analysis and calculated the age-adjusted ROR of the identified disproportional sAEs. The adjusted ROR of the sAEs (Table 2) showed similar patterns, where the most disproportional reactions were skin toxicities (3.4; 95%CI, 2.9–4.0) including Stevens–Johnson syndrome (10.4; 95%CI, 4.0–26.9) with V+C, pyrexia (1.9; 95%CI, 1.5–2.4) and elevated CRP (2.3; 95%CI, 1.2–4.8) with D+T, and renal disorders NEC (4.1; 95%CI, 1.3–12.5) and neurological disorders (peripheral neuropathies—2.7; 95%CI, 1.2–6.1 and seizures 3.8; 95%CI, 1.8–8.0) with E+B. Albeit rare, the adjusted ROR for GBS (8.5; 95%CI, 2.1–35) was substantially higher compared to V+C (0.9; 95%CI, 0.2–4.2) and D+T (0.2; 95%CI, 0.1–0.8). Colitis was also significantly higher (3.3; 95%CI, 1.5–7.1) with E+B; however, the adjusted ROR for GI motility conditions (diarrhea) for E+B (1.8; 95%CI, 1.2–2.6) was slightly lower compared to D+T (2.0; 95%CI, 1.6–2.6) after adjusting for age. 

### 3.4. Clinical Characteristics Using BRAFi and MEKi Combinations

We described the clinical characteristics of cases with sAEs occurring in patients treated with BRAFi+MEKi (Table 3). Among patients with skin disorders treated with V+C, 9% (19/212) have died versus 24% (57/238) and 14% (4/29) in D+T and E+B, respectively. The onset of dermal toxicities occurred soon after the first V+C administration, with a median time to onset of 9 days (IQR 7–14), compared to 30 (IQR 13–50) and 12 (IQR 2–32) using D+T and E+B, respectively. Most reported serious skin disorders (65%, 154/238) occurred in patients treated with doses below 150/2 mg of D+T compared to 15% (32/212) and 3% (1/29) of patients using low V+C (<960/60 mg) and E+B (<450/45 mg) doses, respectively. Most reports of pyrexia were associated with lower doses of D+T (58%, 281/485) doses compared to V+C (7%, 5/69) and E+B (6%, 2/33), with death occurring in 25% (119/485) with D+T versus 14% (10/69) and 6% (2/33) in V+C and E+B, respectively. Pyrexia occurred earlier using V+C (9; IQR 8–22) compared to D+T (28; IQR 16–52) and E+B (35; IQR 12–122). Peripheral neuropathies induced by BRAFi+MEKi combinations were associated with similar death rates of 11% and 12% using V+C (1/9) and E+B (1/8), respectively, and 6% using D+T (2/32). The median time to onset of peripheral neuropathies occurred earlier in E+B (54; IQR 41–57) versus V+C (237; IQR 154–320) and D+T (107; IQR 44–206); however, there were very few cases with details regarding the timing. There were no clear patterns in drug dosing using V+C and D+T; however, most reported reactions with E+B (75%, 6/8) occurred using the recommended dose. Renal disorders were associated with similar death rates with 20% and 21% of the reports in patients using D+T (22/109) and E+B (6/28), respectively, and 13% using V+C (7/53). Most reports of renal toxicities occurred using the recommend dose of E+B with 54% of patients (15/28) and the treatment showed a similar median time to onset of 17.5 days (IQR 5–54) as compared to V+C with 14 days (IQR 7–80), but shorter than D+T, which occurred at 71 days (IQR 54–138). In most cases with sufficient details, the effects of BRAFi+MEKi exposure were resolved after treatment hold, regardless of the type of sAE or the combination.

## 4. Discussion

Novel therapeutic strategies relying on the selective inhibition of the MAPK pathway have proven to significantly prolong the survival of patients with advanced BRAF-mutated melanoma [1,2,3,4,5,6,7]. However, almost all patients treated with BRAFi+MEKi combination therapy develop AEs, with grade 3–4 AEs occurring in most patients [19]. Since the combination therapy with D+T has been approved for adjuvant use in melanoma patients [7] and the indications for BRAFi+MEKi have been extended to other cancer types [52,53,54], the pool of patients is expanding, and thus more patients are expected to develop life-threatening events. As no specific BRAFi+MEKi combination has shown superiority over the rest, there are no evidence-based standard-of-care recommendations of which therapy to choose in the metastatic setting. Therefore, knowledge of the safety profile can be used to tailor the therapy to patients and diagnose adverse reactions early. To our knowledge, we report the most extensive characterization of sAEs associated with BRAFi+MEKi combinations so far, through analysis of the FAERS pharmacovigilance database.

The results show a significant incidence of skin disorders, fever, and GI conditions associated with BRAFi+MEKi combinations, corresponding to the recognized class effects of the treatment [1,13,19]. A comparison between the combinations shows that skin toxicities and fever are overrepresented in V+C and D+T, respectively, while GI conditions were reported significantly less using D+T. These findings are consistent with safety data from phase III clinical studies [1,2,3,4,5,6,19], and corroborate our disproportionality analysis.

Confirmatory studies are necessary to determine therapeutic activity but are limited in providing an accurate depiction of the safety profile, as the conditions under which medicines are used post-marketing may not be reflected in clinical trials, and they also lack sufficient power to detect infrequent or rare events [55,56]. Pharmacovigilance surveillance thus remains the cornerstone for identifying drug-related complications in spite of recognized drawbacks [57]. Herein, we report renal toxicities, neurological disorders and hypotension as additional possible safety considerations for clinicians involved in the care of patients treated with BRAFi+MEKi therapy, and specifically with E+B combination. Data on renal toxicities from V+C and D+T are relatively sparse [2,4,26,58,59] and the association with E+B is even less clear [19,60,61]. We show that nephrotoxicity associated with E+B is significantly more disproportionate among BRAFi+MEKi and portends poor outcomes. Serious renal disorders can occur as early as 17.5 days (IQR 5–54) after initial exposure to E+B. Although the incidence of these renal toxicities cannot be established with the FAERS, some data are available. In the COLUMBUS study, 3% of grade 3–4 AEs encompass renal toxicities [5], which is similar to the proportion reflected in our results of 3.5% (n = 34). Of note, despite having longer follow-up durations, only 1% (grade 3–4 AE) and 0% (any grade AE) of the events were reported in phase III studies for V+C [4] and D+T [62], respectively. This pattern agrees with our findings and provides further support for our results. Furthermore, our report includes the identification of numerous cases with serious peripheral neuropathies and seizures associated with BRAFi+MEKi, which is, to our knowledge, the largest collection of such reports to date. Compared to the full database and BRAFi+MEKi combinations, E+B was associated with a notable reporting odds ratio of peripheral neuropathies and specifically GBS, representing 0.5% of the reports. Nonetheless, previous treatment with immune checkpoint inhibitors (ICIs) cannot be ruled out as a possible contributor or the source for GBS. Several lines of evidence support a major role of MAPK inhibitors as well as encorafenib in the pathophysiology of neurological disorders and GBS. First, the MAPK pathway mediates the dedifferentiation of Schwann cells in the presence of normal axonal signaling and required during the repair process following nerve injury [63]. In addition, the MAPK signaling pathway was found to be significantly enriched in overexpressed genes among patients with GBS [64]. Second, increasing number of case reports and case series were published in the literature, implicating BRAFi or MEKi in neuropathy [65,66,67,68,69,70,71] as well as GBS [69,72,73]. Third, a relatively frequent drug-related AE occurring with encorafenib monotherapy was transient Bell’s palsy [5,18], a mononeuritic variant of GBS, in most cases [74]. This reaction appeared in 8% of the patients and 11% in the dose expansion phase using encorafenib, but has rarely been reported in association with other BRAFi [18]. Facial paresis has also been reported using the combination E+B, but was considerably decreased [5], which can be explained by the suppression of the paradoxical activation of the MAPK pathway [24].

Few limitations of disproportionality analysis need to be recognized. Pharmacovigilance studies have complementary strengths in signal detection that require further validation, optimally in prospective trials. Replication of safety issues in other continental reporting agencies such as the WHO pharmacovigilance database and European Medicine Agency is biased by overlapping safety reports between databases [57]. Further, not all sAEs are submitted to the FAERS, and thus, some reports could be underreported or misrepresented. However, the FAERS provides a key advantage in signal detection, especially of less frequent events, such as GBS, due to the extensive collection of reports from 190 countries, according to the dataset utilized in this study.

One of the major limitations of the analysis involves the inability to quantify the incidence of identified risks, as the total number of patients exposed to a given product is unknown [75]. Further, the background reporting rate could be inflated by an event associated with other drugs, thereby masking the detection of a suspected drug [76]. This phenomenon tends to reduce the sensitivity of some events [77]. Inherent weakness involving the FAERS as well as other spontaneous reporting systems is the inability to verify the clinical findings to justify the reported diagnosis and access relevant data, such as co-morbid illness. Further, a lack of accurate information regarding the line of treatment limits the interpretation of the results. Previous treatment with ICIs could have contributed to the observed toxicities, such as hyperthyroidism which is a common immune-related AE [78]. Furthermore, inconsistent reporting results in missing information regarding demographics, time to onset of AE, drug dosing, dechallenge, rechallenge and comedications. Yet, many successful efforts exist, showing the value of disproportionality to detect safety signals after approval, including examples as early as in the 1980s, where a significant association was found between valproic acid use during pregnancy and spina bifida aperta [79]. Nonetheless, a risk still exists that disproportionality analysis of post-marking data could be misleading. An example of another validated association involves the identification of cefaclor to induce serum sickness-like syndrome [80] and the association between rofecoxib and thrombotic reactions [81]. This relationship, among others, which could have been predicted soon after marketing and long before withdrawal [81], demonstrates the ability to find signals quickly after drug approval [82,83]. Although causality cannot be established conclusively based on post-marketing data alone, it is often served as a basis for adjudicating relationships with sufficient certainty in pharmacovigilance settings [84].

In conclusion, our study shows that beyond skin disorders and fever associated with V+C and D+T, respectively, BRAFi+MEKi therapy can cause other combination-specific toxicities that include gastrointestinal, renal and neurological disorders associated with E+B. Most of the serious adverse reactions commence soon after administration and resolve after dechallenge. These complications should be considered in therapeutic decision making, and careful monitoring is recommended.

## Figures and Tables

**Table 1:** Characteristics of patients with BRAF+MEK-associated adverse events.

		Vemurafenib+Cobimetinib	Dabrafenib+Trametinib	Encorafenib+Binimetinib
Patients		584	1893	350
Reports, No. (%)		2345 (20)	8411 (71.8)	965 (8.2)
Age, Mean (SD), y		58.8 (14.05)	59.4 (16)	62.93 (12.96)
Sex, No. (%)				
	Female	276 (47.26)	789 (41.68)	24 (6.86)
	Male	275 (47.09)	943 (49.82)	26 (7.43)
	Not reported	33 (5.65)	161 (8.51)	300 (85.71)
Indications, No. (%)				
	Melanoma	480 (80.9)	1219 (62.6)	204 (57.8)
	Lung cancer	3 (0.5)	111 (5.7)	4 (1.1)
	Gastrointestinal cancer	4 (0.7)	29 (1.5)	48 (13.6)
	Thyroid cancer	20 (3.4)	34 (1.7)	2 (0.6)
	Hematological malignancy	18 (3)	17 (0.9)	1 (0.3)
	Other/Unspecified	71 (12)	542 (27.8)	96 (27.2)
Country, No. (%)				
	Americas	389 (16.59)	1695 (20.15)	600 (62.18)
	Europe	1570 (66.95)	3389 (40.29)	233 (24.15)
	Australia	33 (1.41)	178 (2.12)	19 (1.97)
	Asia	70 (2.99)	1097 (13.04)	41 (4.25)
	Africa	19 (0.81)	10 (0.12)	0 (0)
	Other	264 (11.26)	2032 (24.16)	72 (7.46)

**Table 2:** Age-adjusted reporting odds ratio for selected serious adverse events.

	V/C Reports (*n* = 2345)	D/T Reports (*n* = 8411)	E/B Reports (*n* = 965)	Reports in Full Database; (*n* = 3,285,265)	Adj. ROR ^1^ (95% CI) V+C vs. D+T and E+B	Adj. ROR ^1^ (95% CI) D+T vs. V+C and E+B	Adj. ROR ^1^ (95% CI) E+B vs. V+C and D+T
Epidermal and dermal conditions	311 (13.3%)	355 (4.2%)	47 (4.9%)	303188 (9.2%)	3.4 (2.9–4.0) ^†^	0.4 (0.3–0.4) ^†^	0.8 (0.6–1.1)
Stevens–Johnson syndrome	17 (0.7%)	6 (0.1%)	0 (0%)	7099 (0.2%)	10.4 (4–26.9) ^†^	0.1 (0.1–0.3) ^†^	
Pyrexia	67 (2.9%)	472 (5.6%)	32 (3.3%)	75,155 (2.3%)	0.5 (0.4–0.6) ^†^	1.9 (1.5–2.4) ^†^	0.7 (0.5–1.1)
Increased CRP	7 (0.3%)	58 (0.7%)	2 (0.2%)	13,829 (0.4%)	0.4 (0.2–0.9)	2.3 (1.2–4.8) ^†^	0.6 (0.1–2.6)
Renal disorders NEC	5 (0.2%)	8 (0.1%)	6 (0.6%)	20,466 (0.6%)	1.7 (0.6–4.7)	0.3 (0.1–0.8) ^†^	4.1 (1.3–12.5) ^†^
Renal disorders (excl. nephropathies)	61 (2.6%)	115 (1.4%)	28 (2.9%)	335,796 (10.2%)	1.8 (1.3–2.4) ^†^	0.5 (0.4–0.7) ^†^	1.8 (1.2–2.9) ^†^
GI motility and defecation conditions	91 (3.9%)	155 (1.8%)	38 (3.9%)	151,750 (4.6%)	2 (1.6–2.6) ^†^	0.5 (0.4–0.6) ^†^	1.8 (1.2–2.6) ^†^
Colitis	13 (0.6%)	19 (0.2%)	12 (1.2%)	8681 (0.3%)	1.9 (1–3.7)	0.3 (0.2–0.6) ^†^	3.3 (1.5–7.1) ^†^
Nausea	40 (1.7%)	127 (1.5%)	29 (3%)	86,674 (2.6%)	1 (0.7–1.5)	0.7 (0.5–1.0)	2.3 (1.4–3.7) ^†^
Seizure	2 (0.1%)	31 (0.4%)	14 (1.5%)	25,004 (0.8%)	0.2 (0–0.8) ^†^	0.9 (0.5–1.6)	3.8 (1.8–8.0) ^†^
Peripheral neuropathies	16 (0.7%)	35 (0.4%)	9 (0.9%)	35,676 (1.1%)	1.3 (0.8–2.4)	0.6 (0.3–0.9)	2.7 (1.2–6.1) ^†^
Guillain–Barre syndrome	2 (0.1%)	4 (0%)	5 (0.5%)	1374 (0%)	0.9 (0.2–4.2)	0.2 (0.1–0.8)	8.5 (2.1–35.0) ^†^
Hypotension	11 (0.5%)	49 (0.6%)	12 (1.2%)	57,946 (1.8%)	0.7 (0.4–1.3)	0.9 (0.5–1.4)	2.5 (1.2–5.1) ^†^

CRP—C-reactive protein. NEC—not elsewhere classified. GI—gastrointestinal. ^1^ Age-adjusted; ^†^ Adjusted *p*-value < 0.05.

**Table 3:** Clinical characteristics of patients with selected serious adverse events.

		Dermal Conditions	Body Temp. Conditions	Renal Disorders *	GI Motility and Defac. Conditions	Seizures (Incl. Subtypes)	Periph. Neurop.	Vascular Hypoten. Disorders
**Drug dosing**								
V/C								
	<960/<60 mg	32/212 (15%)	5/69 (7%)	7/53 (13%)	12/71 (17%)	3/14 (21%)	1/9 (11%)	2/16 (12%)
	960/60 mg	67/212 (32%)	25/69 (36%)	18/53 (34%)	19/71 (27%)	3/14 (21%)	2/9 (22%)	6/16 (38%)
	>960/>60 mg	26/212 (12%)	9/69 (13%)	6/53 (11%)	9/71 (13%)	1/14 (7%)	3/9 (33%)	2/16 (12%)
	Unreported	87/212 (41%)	30/69 (43%)	22/53 (42%)	31/71 (44%)	7/14 (50%)	3/9 (33%)	6/16 (38%)
D/T								
	<150/<2 mg	154/238 (65%)	281/485 (58%)	58/109 (53%)	80/141 (57%)	27/58 (47%)	11/32 (34%)	29/55 (53%)
	150/2 mg	10/238 (4%)	56/485 (12%)	9/109 (8%)	8/141 (6%)	5/58 (9%)	2/32 (6%)	9/55 (16%)
	>150/>2 mg	7/238 (3%)	31/485 (6%)	12/109 (11%)	8/141 (6%)	2/58 (3%)	1/32 (3%)	3/55 (5%)
	Unreported	67/238 (28%)	117/485 (24%)	30/109 (28%)	45/141 (32%)	24/58 (41%)	18/32 (56%)	14/55 (25%)
E/B								
	<450/<45 mg	1/29 (3%)	2/33 (6%)	1/28 (4%)	1/37 (3%)	0/15 (0%)	0/8 (0%)	0/12 (0%)
	450/45 mg	9/29 (31%)	14/33 (42%)	15/28 (54%)	16/37 (43%)	6/15 (40%)	6/8 (75%)	7/12 (58%)
	>450/>45 mg	3/29 (10%)	7/33 (21%)	2/28 (7%)	7/37 (19%)	1/15 (7%)	0/8 (0%)	1/12 (8%)
	Unreported	16/29 (55%)	10/33 (30%)	10/28 (36%)	13/37 (35%)	8/15 (53%)	2/8 (25%)	4/12 (33%)
**Time to AE onset, days (IQR)**								
	V/C	9 (7–14); n = 138	9 (8–22); n = 42	14 (7–80); n = 35	9 (5–27); n = 39	37 (11–185); n = 9	237 (154–320); n = 2	9 (7–13); n = 8
	D/T	30 (13–50); n = 20	28 (16–52); n = 88	70.5 (54–138); n = 24	23 (10–50); n = 19	141.5 (22–303); n = 8	107 (44–206); n = 7	11 (6–76); n = 12
	E/B	12 (2–32); n = 5	34.5 (12–122); n = 14	17.5 (5–54); n = 12	21 (12–27); n = 13	124 (64–128); n = 3	54 (41–57); n = 3	54 (14–131); n = 5
**Dechallenge**								
	V/C	123/134 (92%)	38/44 (86%)	34/36 (94%)	25/27 (93%)	2/2 (100%)	1/1 (100%)	11/11 (100%)
	D/T	116/120 (97%)	239/251 (95%)	60/64 (94%)	57/60 (95%)	15/16 (94%)	10/12 (83%)	32/33 (97%)
	E/B	7/8 (88%)	17/17 (100%)	10/11 (91%)	14/16 (88%)	0/1 (0%)	1/2 (50%)	4/5 (80%)
**Rechallenge**								
	V/C	4/9 (44%)	0/2 (0%)	2/2 (100%)	1/2 (50%)			
	D/T	1/2 (50%)	8/19 (42%)	3/3 (100%)	0/1 (0%)			1/1 (100%)
	E/B	2/2 (100%)	2/3 (67%)	1/3 (33%)	2/3 (67%)			0/1 (0%)
**Outcome**								
V/C								
	Death	19/212 (9%)	10/69 (14%)	7/53 (13%)	16/71 (23%)	6/14 (43%)	1/9 (11%)	0/16 (0%)
	Life-threatening	18/212 (8%)	7/69 (10%)	5/53 (9%)	3/71 (4%)	3/14 (21%)	2/9 (22%)	2/16 (12%)
	Hospitalization	202/212 (95%)	68/69 (99%)	49/53 (92%)	65/71 (92%)	13/14 (93%)	7/9 (78%)	16/16 (100%)
	Other	58/212 (27%)	28/69 (41%)	18/53 (34%)	37/71 (52%)	10/14 (71%)	8/9 (89%)	6/16 (38%)
D/T								
	Death	57/238 (24%)	119/485 (25%)	22/109 (20%)	43/141 (30%)	31/58 (53%)	2/32 (6%)	9/55 (16%)
	Life-threatening	22/238 (9%)	48/485 (10%)	18/109 (17%)	6/141 (4%)	2/58 (3%)	5/32 (16%)	7/55 (13%)
	Hospitalization	204/238 (86%)	441/485 (91%)	103/109 (94%)	132/141 (94%)	52/58 (90%)	32/32 (100%)	53/55 (96%)
	Other	140/238 (59%)	269/485 (55%)	65/109 (60%)	89/141 (63%)	43/58 (74%)	20/32 (62%)	31/55 (56%)
E/B								
	Death	4/29 (14%)	2/33 (6%)	6/28 (21%)	2/37 (5%)	2/15 (13%)	1/8 (12%)	4/12 (33%)
	Life-threatening	5/29 (17%)	3/33 (9%)	5/28 (18%)	3/37 (8%)	4/15 (27%)	0/8 (0%)	1/12 (8%)
	Hospitalization	26/29 (90%)	30/33 (91%)	22/28 (79%)	33/37 (89%)	14/15 (93%)	6/8 (75%)	11/12 (92%)
	Other	12/29 (41%)	12/33 (36%)	12/28 (43%)	12/37 (32%)	9/15 (60%)	3/8 (38%)	5/12 (42%)

* Renal disorders (excluding nephropathies).

## Supplementary Materials

The following are available online at https://www.mdpi.com/2072-6694/12/6/1650/s1, Table S1: Information component of high-level adverse events, Table S2: Reporting odds ratio of serious adverse events (detected as signals) reported for BRAK-MEK inhibitors.
