# Supplementary material for: Safety of BRAF+MEK Inhibitor Combinations: Severe Adverse Event Evaluation
Source: Cancers (Basel). 2020 Jun 22;12(6):1650. doi: 10.3390/cancers12061650 (PMC7352287; doi:10.3390/cancers12061650)
Supplement: Supplementary file 1 [file cancers-12-01650-s001.pdf]

Supplementary Materials: Safety of BRAF+MEK Inhibitor Combinations: Severe Adverse Event Evaluation

Tomer Meirson [1,2], Nethanel Asher [1], David Bomze [3] and Gal Markel [1,4,*]

Table S1. Information component of high-level adverse events.

**Adverse event**	**Vemurafenib + Cobimetinib**			**Dabrafenib + Trametinib**			Encorafenib + Binimetinib		
	**N**	**IC**	**FDR**	**N**	**IC**	FDR	N	IC	FDR
Allergic conditions	130	1.42	1.5e-29	116			5		
Anaemias nonhaemolytic and marrow depression	16			72			12	0.36	2.9e-02
Ancillary infectious topics	23	1.9	1.5e-10	11			6	1.38	3.5e-03
Angioedema and urticaria	18	0.84	1.0e-03	32			3		
Anterior eye structural change, deposit and degeneration	5			9	0.49	2.4e-02	0		
Appetite and general nutritional disorders	23	0.27	2.3e-02	97	0.55	7.9e-06	15	1.08	4.0e-04
Autoimmune disorders	36	1.24	2.5e-08	47			10	0.81	6.7e-03
Bile duct disorders	8	2.23	2.5e-05	4			0		
Body temperature conditions	72	1.3	1.9e-15	496	2.28	1.3e-259	33	1.59	6.0e-11
Central nervous system infections and inflammations	9	1.04	3.3e-03	9			2		
Coagulopathies and bleeding diatheses (excl thrombocytopenic)	2			40	0.83	1.7e-05	0		
Communication disorders and disturbances	9	0.35	3.6e-02	35	0.5	2.2e-03	0		
Cornification and dystrophic skin disorders	5			10	1.82	3.4e-05	6	4.3	1.1e-06
Cutaneous neoplasms benign	8	3.1	3.8e-07	10	1.62	1.1e-04	2		
Deliria (incl confusion)	21	0.74	1.2e-03	46			2		
Eating disorders and disturbances	0			24	0.58	3.0e-03	5		
Electrolyte and fluid balance conditions	67	0.73	1.7e-06	177	0.33	9.5e-05	29	0.94	3.4e-05
Embolism and thrombosis	14			81	0.27	4.9e-03	2		
Endocrine neoplasms malignant and unspecified	0			8	1.88	1.3e-04	0		
Enzyme investigations nec	20	2.23	1.3e-11	122	3.03	1.1e-113	6	1.91	7.0e-04
Epidermal and dermal conditions	311	2.08	6.7e-131	355	0.47	3.7e-11	47	0.77	1.7e-05

Exocrine pancreas conditions	10	0.53	1.9e-02	23			2		
Eye disorders nec	4			23	0.63	2.3e-03	8	2.33	1.7e-05
Fatal outcomes	59			540	0.31	3.4e-08	136	1.54	2.1e-33
Gastrointestinal haemorrhages nec	18			43			10	0.48	2.2e-02
Gastrointestinal infections	14	0.35	2.6e-02	15			1		
Gastrointestinal inflammatory conditions	28	1.34	1.1e-07	50	0.37	3.7e-03	14	1.76	2.3e-06
Gastrointestinal investigations	4			20	2.11	8.1e-11	0		
Gastrointestinal motility and defaecation conditions	91	1.31	7.3e-19	155	0.27	8.6e-04	38	1.46	7.4e-11
Gastrointestinal signs and symptoms	118	0.6	3.4e-07	380	0.48	2.9e-12	68	1.22	5.2e-13
Gastrointestinal ulceration and perforation	12	0.73	6.1e-03	23			6	1.15	6.7e-03
General system disorders nec	249	0.42	2.8e-07	890	0.45	1.7e-21	116	0.74	4.6e-09
Genitourinary tract disorders nec	16	0.71	3.6e-03	44	0.36	5.3e-03	3		
Glaucoma and ocular hypertension	0			6	1.57	2.0e-03	0		
Glucose metabolism disorders (incl diabetes mellitus)	24	0.4	1.1e-02	30			6		
Haematological disorders nec	0			16	1.31	4.0e-05	0		
Haematology investigations (incl blood groups)	8			185	0.78	5.4e-15	11		
Headaches	16			63	0.26	8.2e-03	4		
Heart failures	35	0.1	4.6e-02	97			6		
Hepatic and hepatobiliary disorders	50	0.38	3.3e-03	159	0.25	1.4e-03	19	0.41	1.5e-02
Hepatobiliary investigations	47	1.61	3.4e-15	152	1.5	1.3e-38	10	0.8	6.9e-03
Hepatobiliary neoplasms	1			31	2.14	2.3e-16	1		
Hypothalamus and pituitary gland disorders	6	1.46	2.8e-03	10	0.39	2.9e-02	1		
Immune disorders nec	41	1.07	1.4e-07	90	0.4	3.8e-04	6		
Increased intracranial pressure and hydrocephalus	7	1.82	4.1e-04	25	1.86	5.9e-11	3		
Infections - pathogen unspecified	119	0.15	1.3e-02	362			25		
Injuries by physical agents	11	2.02	3.4e-06	9			0		
Inner ear and viiith cranial nerve disorders	14	1.93	4.1e-07	12			5		
Investigations, imaging and histopathology procedures nec	6	1.39	3.4e-03	5			3		
Lipid metabolism disorders	12	2.14	4.6e-07	11	0.21	4.7e-02	0		

Lymphatic vessel disorders	4			7	1.05	6.2e-03	0		
Malignant and unspecified neoplasms gastrointestinal nec	1			20	0.56	5.3e-03	3		
Metabolism disorders nec	25	1.99	4.9e-12	20			7	1.57	1.0e-03
Metastases	21	2.27	2.0e-12	253	4.05	0.0e+00	6	1.88	7.8e-04
Miscellaneous and site unspecified neoplasms malignant and unspecified	13	0.35	2.8e-02	340	3.25	0.0e+00	20	2.39	1.2e-12
Muscle disorders	53	0.86	8.7e-07	215	1.07	5.0e-29	16	0.55	9.0e-03
Musculoskeletal and connective tissue neoplasms	5			31	2.44	4.5e-20	1		
Myocardial disorders	14	0.78	3.4e-03	33	0.21	2.5e-02	1		
Neoplasm related morbidities	12	2.09	7.0e-07	21	1.1	4.6e-05	4		
Nervous system neoplasms malignant and unspecified nec	2			44	2.97	6.6e-39	6	3.31	1.1e-05
Neurological disorders nec	137	0.11	2.1e-02	441			44		
Neurological disorders of the eye	13	0.97	1.4e-03	50	1.11	3.0e-09	8	1.69	3.2e-04
Neurological, special senses and psychiatric investigations	0			7	0.73	1.6e-02	1		
Neuromuscular disorders	19	0.41	1.4e-02	34			3		
Ocular haemorrhages and vascular disorders nec	0			6	1.43	3.0e-03	0		
Ocular infections, irritations and inflammations	18	1.51	1.8e-06	61	1.47	1.8e-16	4		
Ocular structural change, deposit and degeneration nec	20	3.79	6.7e-24	40	2.99	1.0e-35	7	3.7	3.5e-07
Oral soft tissue conditions	32	1.14	6.7e-07	55	0.12	3.5e-02	8	0.56	2.2e-02
Peripheral neuropathies	16	0.89	1.1e-03	35	0.21	2.4e-02	9	1.48	4.7e-04
Pigmentation disorders	3			15	3.41	3.4e-15	0		
Platelet disorders	7			38	0.36	7.3e-03	1		
Pleural disorders	2			27	0.15	4.0e-02	0		
Protein and amino acid metabolism disorders nec	0			15	1.46	1.7e-05	2		
Protein and chemistry analyses nec	9	0.5	2.3e-02	88	1.99	2.2e-38	3		
Renal and urinary tract investigations and urinalyses	12	0.5	1.7e-02	49	0.73	2.3e-05	10	1.66	9.2e-05
Respiratory and mediastinal neoplasms malignant and unspecified	1			25	1.62	4.6e-09	0		
Respiratory tract neoplasms	2			43	2.07	4.7e-21	1		
Retina, choroid and vitreous haemorrhages and vascular disorders	9	3.8	1.2e-09	3			0		
Salivary gland conditions	10	1.96	1.4e-05	12	0.42	2.2e-02	0		

Seizures (incl subtypes)	15	0.4	1.9e-02	66	0.74	1.6e-06	15	1.82	5.5e-07
Skin and subcutaneous tissue disorders nec	8	0.57	2.1e-02	33	0.82	7.2e-05	0		
Skin and subcutaneous tissue infections and infestations	12	0.52	1.5e-02	58	0.99	8.3e-09	3		
Skin appendage conditions	20	0.95	2.6e-04	52	0.53	4.3e-04	7	0.86	1.1e-02
Skin neoplasms malignant and unspecified	12	1.51	6.5e-05	508	5.11	0.0e+00	14	3.15	8.4e-13
Spinal cord and nerve root disorders	8	1.59	5.0e-04	14	0.59	9.0e-03	2		
Spleen, lymphatic and reticuloendothelial system disorders	4			51	1.6	3.9e-16	1		
Thyroid gland disorders	22	2.5	6.4e-15	29	1.09	3.7e-06	5		
Tissue disorders nec	8	0.47	2.8e-02	15			0		
Tongue conditions	5			21	0.62	3.1e-03	4		
Urinary tract signs and symptoms	3			57	0.25	1.1e-02	2		
Vascular haemorrhagic disorders	38			146			26	0.24	2.7e-02
Vascular inflammations	7	1.83	3.8e-04	4			0		
Vision disorders	22	1.25	5.7e-06	71	1.14	1.6e-12	9	1.38	7.3e-04
Water, electrolyte and mineral investigations	1			60	1.24	1.9e-12	0		

Table S2. Reporting odds ratio of serious adverse events (detected as signals) reported for BRAK-MEK inhibitors.

**Adverse event**	**Vemurafenib + Cobimetinib**			**Dabrafenib + Trametinib**			**Encorafenib + Binimetinib**		
	**N**	**ROR**	**FDR**	**N**	**ROR**	**FDR**	**N**	**ROR**	**FDR**
Abdominal pain upper	7	1.2 (0.5-2.8)	7.2e-01	16	0.4 (0.2-0.9)	1.0e+00	7	3.4 (1.5-8)	8.3e-03
Acute kidney injury	33	2.2 (1.4-3.4)	5.7e-04	51	0.5 (0.3-0.7)	1.0e+00	9	1.2 (0.6-2.4)	6.9e-01
Blood creatinine increased	4	0.4 (0.2-1.2)	1.0e+00	29	1 (0.5-1.9)	1.0e+00	8	2.7 (1.3-5.9)	1.7e-02
C-reactive protein increased	7	0.5 (0.2-1)	1.0e+00	58	2.5 (1.3-5.1)	1.5e-02	2	0.3 (0.1-1.4)	1.0e+00
Colitis	13	1.7 (0.9-3.2)	1.4e-01	19	0.3 (0.2-0.5)	1.0e+00	12	4.2 (2.2-8.2)	6.6e-05
Diarrhoea	68	1.9 (1.4-2.5)	6.0e-05	114	0.4 (0.3-0.6)	1.0e+00	32	2 (1.4-2.9)	8.1e-04
Drug reaction with eosinoph. and systemic symptoms	37	7.9 (4.5-13.8)	2.1e-12	19	0.2 (0.1-0.3)	1.0e+00	0		
Erythema multiforme	15	4.3 (2.1-8.9)	2.3e-04	13	0.3 (0.2-0.7)	1.0e+00	1	0.4 (0.1-2.9)	1.0e+00
Guillain-Barre syndrome	2	0.9 (0.2-4.1)	1.0e+00	4	0.2 (0.1-0.8)	1.0e+00	5	9.3 (2.8-30.6)	5.4e-04
Hyperthyroidism	15	7.5 (3.2-17.8)	1.4e-05	3	0.1 (0-0.2)	1.0e+00	5	3.1 (1.2-8.4)	3.3e-02
Hypotension	11	0.7 (0.4-1.4)	1.0e+00	49	0.8 (0.5-1.4)	1.0e+00	12	2.2 (1.2-4.2)	1.7e-02

Nausea	40	1 (0.7-1.5)	9.0e-01	127	0.7 (0.5-1)	1.0e+00	29	2 (1.3-2.9)	1.8e-03
Pyrexia	67	0.5 (0.4-0.7)	1.0e+00	472	1.9 (1.5-2.4)	3.0e-08	32	0.7 (0.5-0.9)	1.0e+00
Rash	60	2.7 (1.9-3.7)	2.8e-08	79	0.4 (0.3-0.6)	1.0e+00	12	1 (0.5-1.7)	1.0e+00
Rash generalised	17	9.8 (4-23.6)	1.8e-06	7	0.2 (0.1-0.4)	1.0e+00	0		
Rash maculo-papular	29	3.5 (2.1-5.9)	3.1e-06	27	0.3 (0.2-0.5)	1.0e+00	6	1.2 (0.5-2.8)	7.4e-01
Renal disorder	4	1.6 (0.5-5.1)	4.9e-01	5	0.2 (0.1-0.7)	1.0e+00	5	6.2 (2.1-18.6)	2.0e-03
Renal failure	7	0.9 (0.4-2.1)	1.0e+00	24	0.7 (0.3-1.3)	1.0e+00	7	2.5 (1.1-5.8)	3.5e-02
Renal impairment	4	0.9 (0.3-2.6)	1.0e+00	13	0.6 (0.2-1.3)	1.0e+00	5	3.3 (1.2-8.9)	2.7e-02
Seizure	2	0.2 (0-0.7)	1.0e+00	31	0.8 (0.4-1.4)	1.0e+00	14	4.8 (2.6-9)	4.2e-06
Stevens-Johnson syndrome	17	11.4 (4.5-29)	1.5e-06	6	0.1 (0.1-0.4)	1.0e+00	0		
Vertigo	14	3.7 (1.8-7.8)	8.1e-04	10	0.2 (0.1-0.4)	1.0e+00	5	2.3 (0.9-6.1)	1.1e-01
